# Clinical manifestations, risk factors, and prognostic factors of cytomegalovirus enteritis

**DOI:** 10.1186/s13099-021-00450-4

**Published:** 2021-08-18

**Authors:** Pai-Jui Yeh, Cheng-Tang Chiu, Ming-Wei Lai, Ren-Chin Wu, Chien-Ming Chen, Chia-Jung Kuo, Jun-Te Hsu, Ming-Yao Su, Wei-Pin Lin, Tsung-Hsing Chen, Puo-Hsien Le

**Affiliations:** 1 Department of Pediatric Gastroenterology, Chang Gung Memorial Hospital, Linkou Branch, Taoyuan, Taiwan; 2 Department of Gastroenterology and Hepatology, Chang Gung Memorial Hospital, Linkou Branch, Taoyuan, Taiwan; 3 Taiwan Association of the Study of Small Intestinal Disease, Taoyuan, Taiwan; 4 Liver Research Center, Chang Gung Memorial Hospital, Linkou Branch, Taoyuan, Taiwan; 5 Department of Pathology, Chang Gung Memorial Hospital, Linkou Branch, Taoyuan, Taiwan; 6 Department of Medical Imaging and Interventions, Chang Gung Memorial Hospital, Linkou Branch, Taoyuan, Taiwan; 7 Department of General Surgery, Chang Gung Memorial Hospital, Linkou Branch, Taoyuan, Taiwan; 8 Department of Gastroenterology and Hepatology, New Taipei City Municipal Tucheng Hospital (Chang Gung Memorial Hospital, Tucheng Branch), New Taipei City, Taiwan

**Keywords:** Cytomegalovirus enteritis, Endoscopy, Risk factor, Prognostic factor, Immunohistochemistry staining

## Abstract

**Background:**

To comprehensively analyze the risk factors, clinical characteristics, outcomes, and prognostic factors of Cytomegalovirus (CMV) enteritis.

**Methods:**

This retrospective cohort study enrolled patients who had undergone pathological examinations for CMV enteritis. They were divided into CMV and non-CMV groups according to immunohistochemistry staining results. The risk factors, clinical presentations, endoscopic features, treatments, outcomes, and prognostic factors of CMV enteritis were then analyzed.

**Results:**

Forty-two patients (18 CMV, 24 non-CMV group) were included in the study. Major clinical presentations in the CMV group included gastrointestinal bleeding (72.2%), abdominal pain (55.6%), and fever (33.3%); ulcers (72.2%) were the most common endoscopic findings. In-hospital and overall mortality rates were 27.8% and 38.9%, respectively; and longer hospital stays and higher overall mortality rates were observed. Radiotherapy and C-reactive protein levels were prognostic factors for in-hospital mortality. The risk factors for CMV enteritis included immunocompromised status (p = 0.013), steroid use (p = 0.014), shock (p = 0.031), concurrent pneumonia (p = 0.01), antibiotic exposure (p < 0.001), radiotherapy (p = 0.027), chronic kidney disease (p = 0.041), and CMV colitis (p = 0.031).

**Conclusions:**

Physicians should pay attention to the characteristics of CMV enteritis in high-risk patients to make an early diagnosis and potentially improve the clinical outcome.

**Supplementary Information:**

The online version contains supplementary material available at 10.1186/s13099-021-00450-4.

## Background

Cytomegalovirus (CMV) is a double-stranded DNA virus that has a global seroprevalence rate of up to 83% in the general population [1]. It causes not only subclinical infections but also serious tissue invasive diseases. The gastrointestinal (GI) tract is a common location for CMV infection, both in immunocompromised as well as immunocompetent patients; it has variable clinical presentations and a high mortality rate [2–5]. Research of small intestinal CMV disease is relatively rare compared to other GI tract CMV diseases possibly due to difficult tissue sampling.

Prior studies on CMV enteritis were limited to case reports or small case series that established the diagnosis by serological tests and did not analyze small intestinal CMV disease separately [6–11]. For example, Kusne et al. reported 21 episodes of CMV enteritis after intestinal transplantation, but the diagnosis was established by histopathology or virology methods [12]. A literature review of CMV GI diseases in immunocompetent hosts has listed seven cases with small intestine involvement where the diagnosis was confirmed by either biopsy or serological tests, with no detailed clinical or endoscopic information provided [7]. Peter et al. reported the endoscopic findings of cytomegalovirus infection of the upper gastrointestinal tract in solid organ transplant recipients, but the clinical presentations and endoscopic features were statistically analyzed along with CMV gastritis [13]. The latest single-center study reported the characteristics, clinical manifestations, prognosis, and factors associated with gastrointestinal cytomegalovirus infection in immunocompetent patients, but did not analyze CMV small intestinal disease separately [11]. To the best of our knowledge, no study has comprehensively analyzed small intestine CMV disease, including the risk factors, clinical manifestations, endoscopic features, diagnosis, treatments, outcomes, and prognostic factors.

## Methods

### Declarations

The study protocol was approved by the Institutional Review Board of the Chang Gung Medical Foundation on May 14, 2020 for the period of May 01, 2020–May 01, 2021. The Institutional Review Board did not require signed informed consent from individual patients to review medical records from the electronic medical record system, in retrospective studies. The study protocol conformed to the ethical guidelines of the 1975 Declaration of Helsinki, as reflected in a prior approval by the institution’s human research committee.

### Patients

**Fig. 1:**
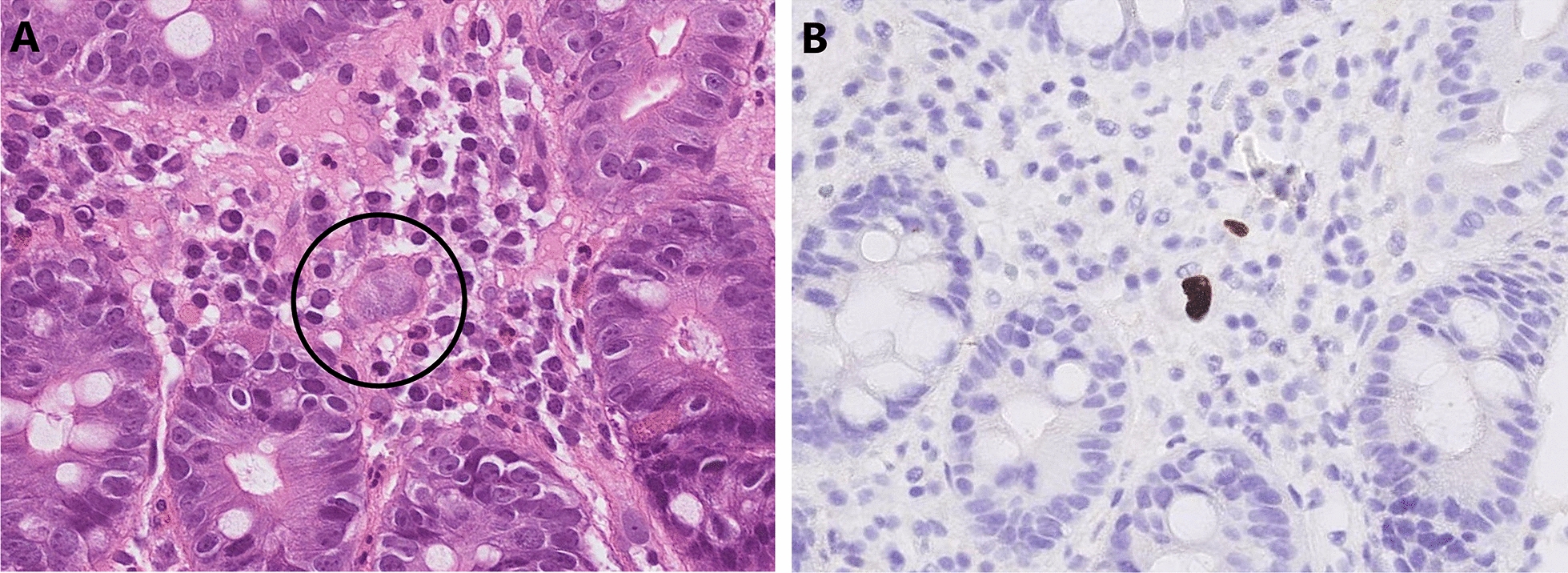
Pathological presentations of CMV enteritis. CMV enteritis was diagnosed using CMV inclusion bodies and IHC staining of the enteric tissue. **A** H&E staining (×40 objective) showing typical intranuclear (owl’s eye) and intracytoplasmic (eosinophilic punctiform) CMV inclusions within the circles. **B** IHC staining (×20 objective) was performed with 1:200 diluted Novocastra™ lyophilized mouse monoclonal antibody against CMV pp65 antigen and showed strong focal CMV immunoreactivity with brownish areas. *CMV* cytomegalovirus, *H&E* hematoxylin and eosin, *IHC* immunohistochemistry

In this retrospective cohort study, we enrolled patients from the pathology database at the Linkou Chang Gung Memorial Hospital, who underwent small intestine CMV immunohistochemistry (IHC) staining between January, 2000 and February, 2021. CMV enteritis was diagnosed based on positive CMV IHC staining of the enteric tissue, with or without viral inclusion bodies, using hematoxylin and eosin staining (Fig. 1). CMV IHC was performed using monoclonal antibodies directed against the CMV pp65 antigen (Novocastra™ lyophilized mouse monoclonal antibody; Leica Microsystems, Wetzlar, Germany). The patients were further divided into two groups (CMV and non-CMV) according to the pathological results.

### Data collection

The medical records of eligible patients were reviewed for data on age, sex, patient source (inpatient, outpatient), admission date, diagnostic date, recurrence date, death or last follow-up, presence of critical condition within 1 week before diagnosis (shock, respiratory distress with ventilator usage), underlying disease, medication history, major clinical presentation, endoscopic findings (lesion characteristics, location, number, and concomitant mucosal findings), histopathology results (presence of malignancy or findings indicating other etiology of enteritis), treatment and therapeutic duration, complications, outcomes (in-hospital mortality rate and overall mortality rate), total white blood cell count, absolute neutrophil count, absolute lymphocyte count, platelet count, hemoglobin (Hb), creatinine (Cr), aspartate aminotransferase, alanine aminotransferase, bilirubin, albumin, C-reactive protein (CRP) levels, CMV pp65 antigenemia, CMV DNA (226 bp segment on glycoprotein B gene, LightMix® Kit human cytomegalovirus; TIB Molbiol, Berlin, Germany), and CMV serology.

### Definition of immune status

Patients were defined as “immunocompromised” if they were documented to have primary immunodeficiency, human immunodeficiency virus infection, use of chemotherapeutic agents within 6 months, use of immunosuppressants [including corticosteroids (oral or intravenous administration, ≧ 20 mg/day of prednisolone or any equivalent for > 2 weeks)], or were recipients of solid organ or bone marrow transplantation [3, 11].

### Statistical analyses

Numerical data are presented as mean ± standard deviation or median (interquartile range), while categorical data are expressed as absolute numbers and percentages. Independent t-tests and Mann–Whitney U tests were used to compare continuous variables, while χ^2^ and Fisher’s exact tests were used for categorical variables. Logistic regression models were used to identify the independent risk factors for in-hospital mortality. Statistical significance was set at p < 0.05. The results are presented as odds ratios (ORs), 95% confidence intervals (CIs), and p-values. Survival outcomes were evaluated using Kaplan–Meier survival curve analysis and log-rank test. All statistical calculations were performed using the SPSS statistical software, version 21.0 (Armonk, NY: IBM Corp.).

## Results

### Demographics of CMV enteritis

Forty-two patients were enrolled, including 18 in the CMV group and the rest in the non-CMV group. In the CMV group, 15 (83.3%) were in-hospital patients, and four of them needed intensive care. The mean age was 49.8 ± 19.9 years, and males were predominant (66.7%). Most patients with CMV enteritis (77.8%) were immunocompromised. The major comorbidities were chronic kidney disease, malignancy, and hypertension. The types of malignancies were diverse but mostly involved solid organs [lung, brain, bladder, prostate, ileum (adenocarcinoma), liver (cholangiocarcinoma), and skin]. One patient with ileal adenocarcinoma was diagnosed and treated before the development of CMV enteritis. Three patients had undergone renal transplantation (without antiviral prophylaxis), and three had autoimmune diseases (systemic lupus erythematosus, autoimmune hepatitis, and Behcet’s disease). CMV colitis and CMV gastritis (concurrent diagnosis or past history) were noted in six and one patient, respectively. More patients with Crohn’s disease were enrolled in the non-CMV group because physicians always took biopsy specimens from terminal ileal ulcers to confirm the diagnosis of Crohn’s disease and concurrent CMV infection. Other details and differences between the two groups are presented in Table 1.

**Table 1:** Baseline characteristics of the patients with or without CMV enteritis

Characteristics	Overall (n = 42)	CMV enteritis (n = 18)	Non-CMV enteritis (n = 24)	p-value
Age, years	49.7 ± 21.5	49.8 ± 19.9	49.5 ± 23	0.809
Sex (M/F)	24(57.1%)/18(42.9%)	12(66.7%)/6(33.3%)	12(50%)/12(50%)	0.353
General conditions				
Shock	7 (16.7%)	6 (33.3%)	1 (4.2%)	0.031*
Pneumonia	5 (11.9%)	5 (27.8%)	0 (0%)	0.01*
Intubation	5 (11.9%)	4 (22.2%)	1 (4.2%)	0.146
ICU required	6 (14.3%)	4 (22.2%)	2 (8.3%)	0.375
Underlying diseases				
Immunocompromised	23 (54.8%)	14 (77.8%)	9 (37.5%)	0.013*
Diabetes mellitus	4 (9.5%)	1 (5.6%)	3 (12.5%)	0.623
Hypertension	12 (28.6%)	6 (33.3%)	6 (25%)	0.732
Autoimmune disease	3 (7.1%)	3 (16.7%)	0 (0%)	0.071
Coronary artery disease	3 (7.1%)	3 (16.7%)	0 (0%)	0.071
COPD	1 (2.4%)	1 (5.6%)	0 (0%)	0.429
Renal disease				
AKI	3 (7.1%)	3 (16.7%)	0 (0%)	0.071
CKD	13 (31%)	9 (50%)	4 (16.7%)	0.041*
ESRD	6 (14.3%)	5 (27.8%)	1 (4.2%)	0.068
Crohn’s disease	12 (28.6%)	2 (11.1%)	10 (41.7%)	0.042*
Ulcerative colitis	1 (2.4%)	1 (5.6%)	0 (0%)	0.429
HIV infection	1 (2.4%)	1 (5.6%)	0 (0%)	0.429
Malignancies	13 (31%)	8 (44.4%)	5 (20.8%)	0.177
Transplantation	4 (9.5%)	3 (16.7%)	1 (4.2%)	0.297
Antibiotics usage	19 (45.2%)	14 (77.8%)	5 (20.8%)	< 0.001*
Immunosuppressive therapies				
Steroid	28.6 (57.4%)	9 (50%)	3 (12.5%)	0.014*
Chemotherapy	3 (7.1%)	2 (11.1%)	1 (4.2%)	0.567
Radiotherapy	4 (9.5%)	4 (22.2%)	0 (0%)	0.027*
Immunosuppressant	10 (23.8%)	6 (33.3%)	4 (16.7%)	0.281
Laboratory data				
WBC count (/μL)	7200 (5400–9500)	7050 (5050–9750)	7200 (6000–9500)	0.705
Hemoglobin (g/dL)	10.4 (8.1–12.1)	9.2 (7.3–10.7)	11.2 (9.6–12.4)	0.027*
Platelet (× 1000/mm^3^)	231 (152–294)	182 (143.3–275.8)	261 (199–294)	0.175
Creatinine (mg/dL)	1 (0.6–2.1)	1.2 (0.7–4)	0.8 (0.6–1.2)	0.151
ALT (IU/L)	16.5 (13.3–21)	19 (15–23.8)	15.5 (10–18.5)	0.05
Albumin (g/dL)	3.3 (2.5–3.6)	2.9 (2.4–3.3)	3.6 (3.2–4)	0.067
C-reactive protein (mg/dL)	30 (16.7–115.3)	81.3 (24.8–153.3)	21.5 (6.3–73.8)	0.027*
Virology/serology positivity				
CMV-IgM	3/14 (21.4%)	3/10 (30%)	0/4 (0%)	0.506
CMV-IgG	12/14 (85.7%)	10/10 (100%)	2/4 (50%)	0.066
CMV pp65 antigenemia	3/ 12 (25%)	3/9 (33.3%)	0/3 (0%)	0.509
CMV PCR	5/9 (55.6%)	5/7 (71.4%)	0/2 (0%)	0.167
Clinical presentation				
Fever	7 (16.7%)	6 (33.3%)	1 (4.2%)	0.031*
Abdominal pain	22 (52.4%)	10 (55.6%)	12 (50%)	0.764
Vomiting	9 (21.4%)	5 (27.8%)	4 (16.7%)	0.462
Diarrhea	12 (28.6%)	8 (44.4%)	4 (16.7%)	0.084
Abdominal fullness	11 (26.2%)	5 (27.8%)	6 (25%)	1.000
GI bleeding	20 (47.6%)	13 (72.2%)	7 (29.2%)	0.012*
Endoscopic features				
Main findings				
Ulcer	35 (83.3%)	13 (72.2%)	22 (91.7%)	0.118
Inflammation	5 (11.9%)	2 (11.1%)	3 (12.5%)	1.000
Polypoid lesion	10 (23.8%)	6 (33.3%)	4 (16.7%)	0.281
Location of lesion				
Duodenum	5 (11.9%)	3 (16.7%)	2 (8.3%)	0.636
Jejunum	5 (11.9%)	1 (5.6%)	4 (16.7%)	0.371
Ileum	32 (76.2%)	14 (77.8%)	18 (75%)	1.000
Concurrent findings				
CMV gastritis	1 (2.4%)	1 (5.6%)	0 (0%)	0.429
CMV colitis	7 (16.7%)	6 (33.3%)	1 (4.2%)	0.031*
Outcomes				
Perforation	3 (7.1%)	1 (5.6%)	2 (8.3%)	1.000
Overall mortality	8 (19%)	7 (38.9%)	1 (4.2%)	0.013*
In-hospital mortality	6 (14.3%)	5 (27.8%)	1 (4.2%)	0.068
Hospital stay, days	42 (15–64.5)	35 (22.5–48.5)	8.5 (4–20.5)	0.001*

*AKI* acute kidney injury, *ALT* alanine aminotransferase, *AST* aspartate aminotransferase, *CKD* chronic kidney disease, *CMV* cytomegalovirus, *COPD* chronic obstructive pulmonary disease, *ESRD* end-stage renal disease, *F* female, *GI* gastrointestinal, *ICU* intensive care unit, *IQR* interquartile range, *M* male, *SD* standard deviation, *WBC* white blood cell

*P < 0.05. Age is presented as mean ± standard deviation. Laboratory data, hospital stay, and follow-up duration are presented as median (IQR). The remaining data were presented as numbers (percentages)

### Clinical manifestations of CMV enteritis

The major symptoms of CMV enteritis include GI bleeding (more hematochezia than melena), abdominal pain, diarrhea, and fever. Only one patient presented with intestinal perforation at the time of diagnosis: this 4-year-old boy with newly diagnosed Crohn’s disease developed ileal perforation after receiving around 2 weeks of corticosteroids.

### Diagnosis of CMV enteritis

Anemia, hypoalbuminemia, and elevated CRP levels were noted in patients with CMV enteritis. As for virology tests, all the patients were positive for CMV-IgG, however, only around one-third were positive for either CMV-IgM, viremia, or CMV pp65 antigenemia.

**Fig. 2:**
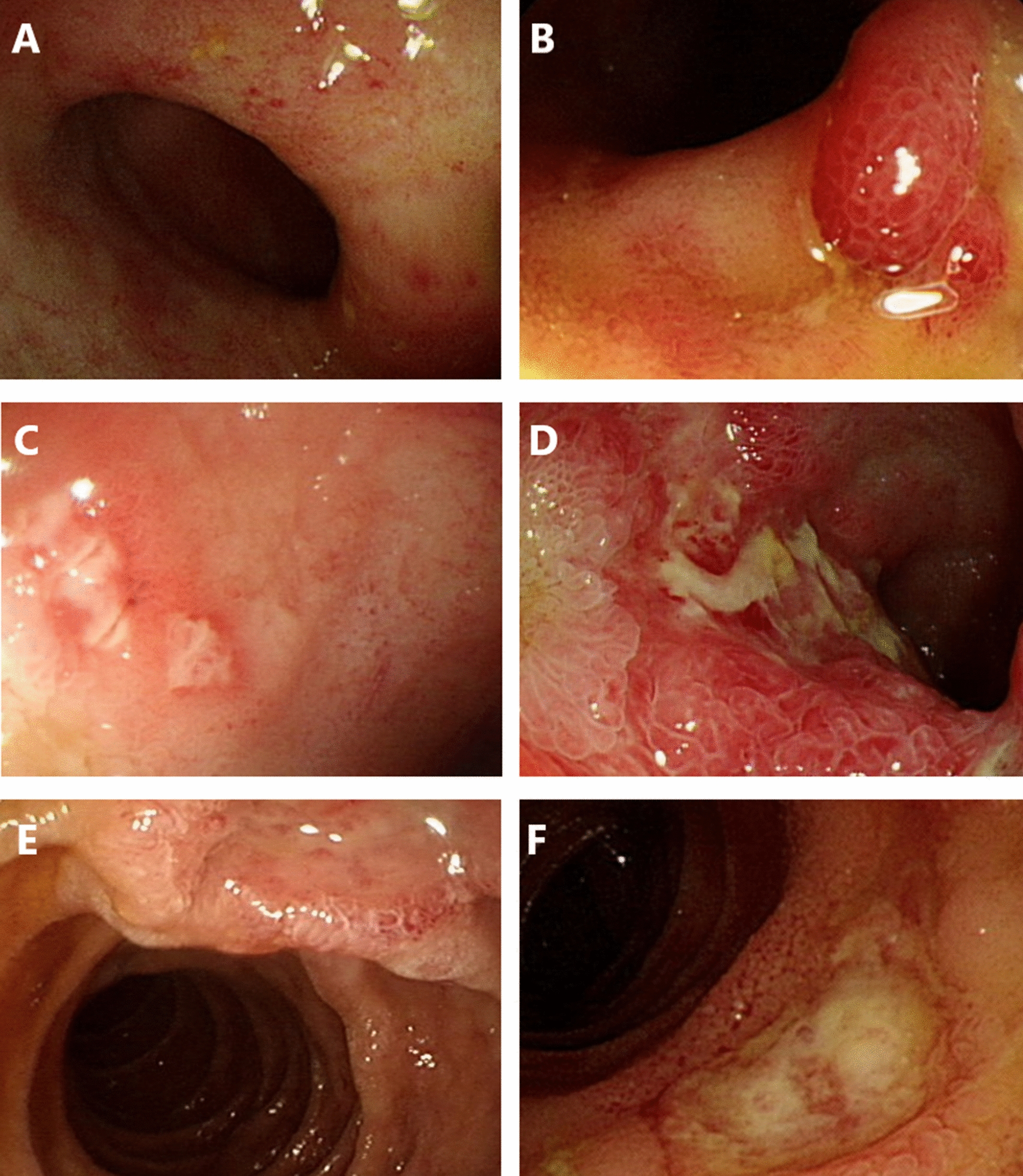
Endoscopic features of CMV enteritis. **A** Inflammation. **B** Polypoid lesion. **C**–**F** Variable morphologies of enteric ulcers. *CMV* cytomegalovirus

Regarding the methods of tissue sampling, colonoscopy (in 11 patients, including 1 with sigmoidoscopy) and esophagogastroduodenoscopy (in 3 patients) were the two major modalities, while per-anus double balloon enteroscopy was performed in 2 patients. Before the index endoscopy, which confirmed the diagnosis, eight patients (44.4%) had undergone more than one endoscopic examination, indicating difficulty in confirmation of the diagnosis, resulting in the need for repeated approaches. Endoscopically, ulcers were the leading presentation, followed by polypoid lesions and inflammation (in the absence of ulcer or mass-like change) (Fig. 2). Regarding location, the ileum was the most frequently involved segment. Histopathological samples from five patients with CMV enteritis did not show typical CMV viral inclusion bodies, but atypical lymphocytes or prominent germinal centers with inflammatory infiltrates.

In this study, the mean time to diagnosis [from admission (or first visit to the outpatient clinic) to diagnosis of CMV enteritis] was 18.9 ± 15.8 days, ranging from 2 to 63 days.

### Treatments and outcomes of CMV enteritis

In total, 12 patients (66.7%) received antiviral therapy (intravenous ganciclovir or oral valganciclovir), with a variable duration range (intravenous: 6–23 days, oral: 7–194 days). Three patients underwent surgeries for CMV enteritis to bypass the stricture or repair the intestinal perforation. The mean duration of admission was 35.8 ± 15.6 days, and the median follow-up duration was 58 (35–209) days. The in-hospital and overall mortality rates were 27.8% and 38.9%, respectively. No disease recurrence was observed during the follow-up period.

### Prognostic factors associated with the in-hospital mortality of CMV enteritis

History of radiotherapy (OR, 18; 95% CI 1.194–271.461; p = 0.037) and CRP levels (OR, 1.028; 95% CI 1.001–1.057; p = 0.045) were the only two independent prognostic factors for in-hospital mortality (Table 2). In the Kaplan–Meier survival curve analysis, patients with CMV enteritis having a history of radiotherapy had significantly poorer survival rates than those without a history of radiotherapy (log-rank p = 0.013) (Additional file 1: Figure S1). Antiviral therapy (OR, 2.5; 95% CI 0.214–29.254; p = 0.465) did not significantly influence the survival outcome.

**Table 2:** Analysis of clinical factors associated with in-hospital mortality

Characteristics	Univariable analysis		
	OR	95% CI	p-value
Age	1.308	0.97–1.111	0.285
Sex (male)	0.667	0.078–5.678	0.711
Time to diagnosis	1.031	0.966–1.1	0.365
General conditions			
Shock	5	0.551–45.391	0.153
Pneumonia	2.222	0.245–20.174	0.478
Intubation	3.667	0.354–38.029	0.276
ICU required	3.667	0.354–38.029	0.276
Immunocompromised	897,486,006	0	0.999
Underlying diseases			
Diabetes mellitus	0	0	1
Hypertension	0.4	0.034–4.681	0.465
Autoimmune	1.375	0.096–19.643	0.814
Acute kidney injury	1.375	0.096–19.643	0.814
Chronic kidney disease	1.75	0.215–14.224	0.601
End stage renal disease	0.563	0.047–6.769	0.65
Malignancy	9	0.748–108.31	0.083
Transplantation	0	0	0.999
Steroid	1.75	0.215–14.224	0.601
Chemotherapy	3	0.15–59.89	0.472
Radiotherapy	18	1.194–271.461	0.037*
Laboratory data			
WBC count	1	1	0.39
Hemoglobin	0.76	0.496–1.163	0.206
Platelet	0.994	0.983–1.006	0.362
Creatinine	1.046	0.665–1.645	0.845
ALT	0.966	0.885–1.053	0.432
Albumin	0.202	0.017–2.39	0.205
C-reactive protein	1.028	1.001–1.057	0.045*
Clinical symptoms			
Fever	1.5	0.176–12.775	0.711
Abdominal pain	4.667	0.404–53.95	0.217
Vomiting	8.25	0.795–85.564	0.077
Diarrhea	0.778	0.096–6.322	0.814
Abdominal fullness	2.222	0.245–20.174	0.478
GI bleeding	1.778	0.148–21.395	0.65
Endoscopic features			
Ulcer	1.778	0.148–21.395	0.65
Inflammation	3	0.15–59.89	0.472
Polypoid lesion	0	0	0.999
Duodenum	1.375	0.096–19.643	0.814
Jejunum	5,250,293,239	0	1
Ileum	0.273	0.026–2.829	0.276
Antiviral treatment	2.5	0.214–29.254	0.465

*ALT* alanine aminotransferase, *CI* confidence interval, *F* female, *GI* gastrointestinal, *ICU* intensive care unit, *M* male, *OR* odds ratio, *WBC* white blood cell

*P < 0.05, calculated by logistic regression analysis

### Comparison between CMV and non-CMV groups

More patients in the CMV group had an immunocompromised status, chronic kidney disease, shock, pneumonia, and a history of CMV colitis than those in the non-CMV group. Moreover, a higher proportion of patients in the CMV group had exposure to steroids, antibiotics, and radiotherapy than those in the non-CMV group. Clinically, the CMV group had a higher prevalence of GI bleeding and fever, than the non-CMV group, which contributed to lower Hb levels and higher CRP levels. With respect to outcomes, patients in the CMV group had significantly longer admission duration and higher overall mortality rate than those in the non-CMV group.

## Discussion

CMV enteritis is a rare but fatal disease that has been under diagnosed. It is not only an opportunistic infection in immunocompromised patients, but also affects immunocompetent individuals [2, 3, 10, 14]. Despite the development of small intestinal endoscopies, such as capsule endoscopy, single-balloon enteroscopy, and double-balloon enteroscopy, diagnosis of CMV enteritis is still difficult due to critical general condition of the patients, various clinical presentations, and expensive procedure fees. Eight patients (44.4%) underwent more than one endoscopic examination before the final diagnosis. The time to diagnosis in our series was approximately 3 weeks; however, no similar data were available in the literature for comparison. Nevertheless, these facts imply the importance of repeated endoscopic biopsies in high-risk patients.

In view of histopathological diagnosis of CMV enteritis, a discrepancy between the results of H&E staining and IHC staining has been reported [15]. The specificity and sensitivity of CMV IHC staining has been reported to approach 93% and 100% [14], respectively; however, the cytopathic effects are observed in only about 65% of cells that show positive CMV IHC staining [16]. Therefore, we established positive IHC staining as the diagnosis for CMV enteritis.

Higher CRP levels and history of radiotherapy were the poor prognostic factors for in-hospital mortality. Higher CRP levels represent severe inflammation and infection, leading to a higher in-hospital mortality rate [17]. Radiotherapy can result in acute and chronic bowel damage [18, 19], because ionizing radiations cause inflammation, mucosal cell loss, swelling of the endothelial lining of arterioles, ischemia, and neovascularization, all of which contribute to mucosal friability and fibrosis [20]. In this study, three out of four patients with a history of radiotherapy were irradiated in the pelvic and lower spinal regions, which probably jeopardized their bowel integrity, leading to a worse outcome.

In this cohort study, the clinical features and risk factors of CMV enteritis were analyzed by comparing the CMV and non-CMV groups. In the CMV enteritis group, a significantly higher proportion of patients developed fever and GI bleeding in combination with higher CRP and lower Hb levels, indicating more severe mucosal injury and inflammation, than that in the non-CMV group. The risk factors for CMV enteritis can be categorized into three categories: predisposing mucosal injury (radiotherapy), comorbidities (CMV colitis), and immunodeficiency (immunocompromised status, shock, concurrent pneumonia, corticosteroid exposure, and chronic kidney disease). As stated earlier, radiotherapy leads to acute and chronic mucosal injury, contributing to the vulnerability and risk of superimposed infection. Second, CMV colitis is a risk factor for CMV enteritis because they share similar risk factors; this association was first mentioned in our study. Third, in addition to immunocompromised status, critical illness (shock, concurrent pneumonia), corticosteroid exposure, and chronic kidney disease lead to immunodeficiency [21, 22]. In addition, antibiotics are commonly prescribed for critical conditions, such as severe pneumonia and shock. Therefore, CMV enteritis should be considered when high-risk patients suffer from fever and GI bleeding.

The limitations of this study include the small number of cases, retrospective design, and single-center-based resources. However, it may not be feasible to conduct a prospective study for this rare yet frequently fatal disease.

## Conclusion

This is the first cohort study to demonstrate the comprehensive features of CMV enteritis (small intestine), treatments, outcomes, risk factors, and prognostic factors with scrupulous diagnostic criteria. Radiotherapy, CMV colitis, and immunodeficiency are risk factors for CMV enteritis. Fever and GI bleeding are the most common symptoms. Meanwhile, radiotherapy and higher CRP levels are poor prognostic factors for in-hospital mortality. Physicians should be aware of these characteristics, which could enable them to make an early diagnosis and potentially improve the clinical outcome.

## Supplementary Information

**Additional file 1: Figure S1.** Kaplan–Meier survival curve analysis of CMV enteritis patients with/without radiotherapy. Patients with CMV enteritis who received radiotherapy (black line) had a significantly worse survival rate than those who did not receive radiotherapy (gray line) (log-rank p = 0.013). CMV, cytomegalovirus.

## Data Availability

The datasets used and/or analysed during the current study are available from the corresponding author on reasonable request.
