# Supplementary figures and images for: Clinical manifestations, risk factors, and prognostic factors of cytomegalovirus enteritis
Source: Gut Pathog. 2021 Aug 18;13:53. doi: 10.1186/s13099-021-00450-4 (PMC8371416; doi:10.1186/s13099-021-00450-4)

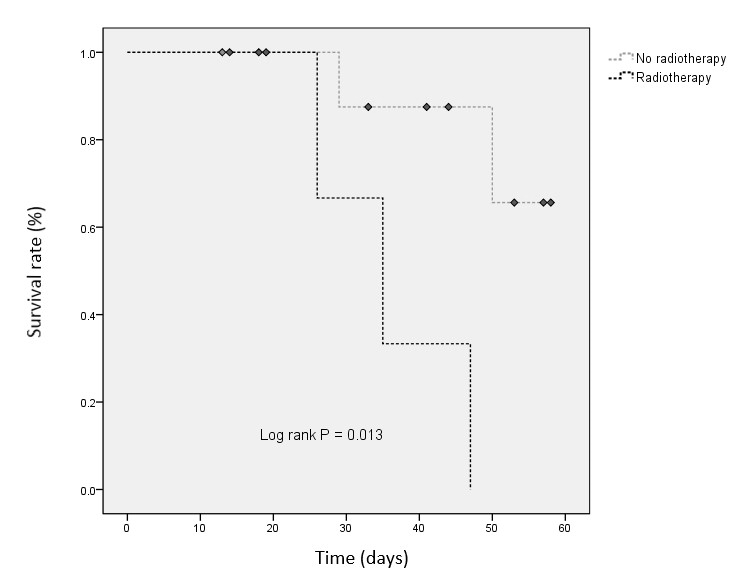

Supplement: Supplementary file 1 — Additional file 1: Figure S1. Kaplan–Meier survival curve analysis of CMV enteritis patients with/without radiotherapy. Patients with CMV enteritis who received radiotherapy (black line) had a significantly worse survival rate than those who did not receive radiotherapy (gray line) (log-rank p = 0.013). CMV, cytomegalovirus. [file 13099_2021_450_MOESM1_ESM.tif]
